# Application of the Polar Coordinate Technique in the Study of Technical-Tactical Scoring Actions in Taekwondo

**DOI:** 10.3389/fspor.2022.877502

**Published:** 2022-05-23

**Authors:** José Antonio Gamero-Castillero, Yarisel Quiñones-Rodríguez, Gennaro Apollaro, Antonio Hernández-Mendo, Verónica Morales-Sánchez, Coral Falcó

**Affiliations:** ^1^Facultad de Psicología, Universidad de Málaga, Málaga, Spain; ^2^Department of Social Psychology, Social Work, Anthropology and East Asian Studies, University of Málaga, Málaga, Spain; ^3^School of Sport Sciences and Exercise, Faculty of Medicine and Surgery, University of Rome “Tor Vergata”, Rome, Italy; ^4^Department of Sport, Food and Natural Sciences, Western Norway University of Applied Sciences, Bergen, Norway

**Keywords:** polar coordinates, observational methodology, taekwondo, observation tool, technical-tactical actions, bout analysis

## Abstract

The objective of this study was to design, validate and update an observation tool to analyse the technical-tactical actions by which taekwondo players win points. An *ad hoc* observational tool was developed for subsequent use in HOISAN software by viewing seven finals (14 viewings) in the Rome 2019 Grand Prix, collecting data (1,382 technical/tactical actions) from both winners and losers (women, *n* = 3; men, *n* = 4). An observational methodology based on a nomothetic, follow-up and multidimensional (N/F/M) observational design was used. In the statistical analysis, to check the validity of the generalizability analysis, the Category/Observer (C/O) and Observer/Category (O/C) models were employed, and to determine reliability between observations (intra- and interobserver), the Pearson, Spearman, Kendall's tau-b and Cohen's kappa correlation coefficients were applied. One point were awarded for every penalty given against the opponent. Two points were obtained for a circular technique to the trunk protector (in winners) or by scoring a point for a punch plus a penalty against the opponent, three points in melee actions, anticipatory actions with the left side (winners), or after a punch action, and circular technique (losers). Four and five points were only obtained by winners, in a direct attack with the right leg, turning (five points) or simultaneous (four points). The results of this study provide information on the most effective technical-tactical actions in taekwondo for scoring points in a contest.

## Introduction

Taekwondo has been an Olympic combat sport since the Sydney 2000 Olympics. A contest or bout consists of three 2-min rounds with a 1-min rest between rounds. If the third round finishes in a tie, a further period is added, with a maximum duration of 1 min, and the first competitor to score two or more points or whose opponent receives two penalties is declared the winner (World Taekwondo, 2020). Since becoming an Olympic sport, taekwondo has undergone several modifications in its scoring system, the latest being in October 2020 (World Taekwondo, 2020). So according to the rules, since June 2018 the valid scoring areas have been as follows: one point (1P) for a valid punch to the trunk protector, two points (2P) for a valid kick to the trunk protector, four points (4P) for a valid turning kick to the trunk protector, three points (3P) for a valid kick to the head, five points (5P) for a valid turning kick to the head, and one point (1P) for each penalty (“Gam-jeom”) given against the opponent.

Observational methodology is suitable for studying the situational factors in a taekwondo contest. Moreover, it is a valid support for undertaking a description of how taekwondo players behave in their natural setting (Anguera et al., 2001; Castellano and Hernández-Mendo, 2003). In observational methodology, interrelated behaviors are important in order for athletes and coaches to be able to find out which types of behavior are dependent or non-existent, while others do occur (Menescardi et al., 2020a). This methodology makes it possible to record and quantify performance objectively, reveal technical and tactical behaviors, and introduce patterns of behavior (Menescardi et al., 2020b). When it comes to creating and using an observation tool, one must take account of the factors that affect athletes' behavior, as well as ensuring that the tool is suited to the scoring system (Menescardi et al., 2020b). The observation tool must be based on prior studies and data to be able to produce an improved and exceptionally valid design, provide precise observations and take the most important performance variables into account (Menescardi et al., 2017). The tool must be exhaustive and mutually exclusive for each criterion; in other words, it must be precise and a behavior cannot be classified in two categories of the tool simultaneously (Anguera-Argilaga et al., 2011; Blanco-Villaseñor et al., 2014; Menescardi et al., 2017). If this requirement is not complied with, the definitions of the categories used in the tool must be reviewed, since it means that the data are not reliable, and moreover they may alter the results of the study erroneously (Anguera et al., 2007; Menescardi et al., 2017). In combat sports, the use of observational methodology is transcendental, mainly to study technical-tactical interactions in competition. In Karate, it has been used to examine technical-tactical actions and identify the regular behavioral structures of elite competitors (Ibáñez et al., 2018). In fencing to analyse the behaviors in competition that consolidate or modify performances during each round (Tarragó et al., 2017), in elite men's foil they study tactical actions and their impact on the scoreboard (Iglesias et al., 2018) and in judo they analyse the technical-tactical patterns that score in bouts at the highest level of competition (Gutiérrez-Santiago et al., 2019).

To date, the scientific literature has shown us various tools (Gutiérrez-Santiago et al., 2020; Menescardi et al., 2020b; Barrientos et al., 2021) validated for the analysis of tactical actions in taekwondo. However, rule changes (the latest was made in 2018), as well as the evolution of athletes and their technical-tactical approaches, make it necessary to update these tools constantly, in order to adapt the observations and analyses based on the resources they deploy. In this connection, Gutiérrez-Santiago et al. (2020) have recently proposed a new taxonomy consisting of 10 criteria and 51 categories, which analyses the arm technique (type of block), the technique used (out of a total of 13), as well as the movement of the attacking leg (in its initiation and landing, out of a total of 5), or the type of change of position (out of a total of 15). Barrientos et al. (2021), in turn, propose a model with five criteria, 31 categories and 96 subcategories. Among these models, we can highlight combined actions and clinches. Combined actions are those in which two or more techniques are performed before the first striking leg touches the floor; these may be combined techniques with the same leg (corrections) or a different leg (double techniques). Clinch actions, for their part, have appeared as a result of the rule change establishing that when the two opponents come into contact, the contest shall not be stopped, provided that one of the athletes is trying to score a point in a permitted area (World Taekwondo, 2010).

One of the various analytical techniques that can be found in observational methodology is polar coordinate analysis (Sackett, 1980), which offers the possibility of estimating the types of relationships that are established between focal and conditioned behaviors (Castellano and Hernández-Mendo, 2003). Polar coordinate analysis originated in the study by Sackett (Sackett, 1980) which is based on a sequential analysis of prospective and retrospective lags, subsequently optimized with the “genuine technique” (Anguera, 1997), which makes it possible to achieve a drastic reduction of the data on the various behaviors that have occurred and a vector representation of the various interrelationships established between the different categories in the taxonomic system (López-López et al., 2015). This type of technique is becoming increasingly important in the world of sport, as polar coordinate analysis along with sequential analysis are characteristic of observational methodology (Anguera and Hernández-Mendo, 2014; López-López et al., 2015).

Few studies have so far analyzed the behaviors of winning and losing taekwondo athletes (Falcó et al., 2014; González-Prado et al., 2015; Menescardi et al., 2019, 2020c). It seems that the participants in six world championships and world cups between 2000 and 2008 scored more points the more offensive actions they performed. In the university sphere, winners perform more counterattacks than losers, and among their attacks and counterattacks there are fewer indirect attacks and more anticipatory counterattacks (Falcó et al., 2014). In the same sample, and in relation to rounds, the results showed that winners perform more anticipatory counterattacks (in the second and third rounds), fewer subsequent actions (in the third round) and a lower number of simultaneous actions in the first and especially in the third round than losers (Menescardi et al., 2015). According to Menescardi et al. (2020c), winners perform more anticipatory actions (scoring one and three points), whereas losers execute a larger number of back-leg, indirect and turning actions.

A study carried out by Menescardi et al. (2019), in which they analyzed the contests of two Olympic medallists over the course of two Olympics (London 2012 and Río 2016), shows that a point was obtained with direct attacks to the trunk protector. After scoring, they continued with an action to the head. Two points were achieved with simultaneous turning actions, in which subsequent actions occurred afterwards, whereas cutting was performed before and after scoring. Three points were obtained with indirect attacks and subsequent counterattacks to the head. Cutting, feinting and linear techniques preceded obtaining three points, whereas feints, direct attacks and linear actions to the trunk protector occurred in succession after three points were scored. In a sample of 12 taekwondo athletes who participated in the 2016 Río Olympics, Menescardi et al. (2020a) found that winners obtained one point by executing a direct attack to the trunk protector and that after scoring they performed a back-leg technique or a cut. Three points were obtained with a turning kick to the head, continuing with a front-leg action. Cutting actions occurred before scoring three points, while direct attacks were executed afterwards. Therefore, taking the rule change as well as previous studies into account (Falcó et al., 2014; Menescardi et al., 2015, 2020b,c) the objective of this article is twofold: firstly to design an observation tool under the new rules (World Taekwondo, 2020), validate it, certify its reliability and update it, and secondly, using a polar coordinate analysis, to identify the technical-tactical actions by which winning and losing taekwondo competitors managed to score in the finals of the Rome 2019 Grand Prix.

## Methods

### Methodology and Study Design

This study was conducted using observational methodology. It involved a nomothetic, follow-up and multidimensional (N/F/M) observational design to study all the participants' movements, taking account of their behaviors and the dimensions matching the criteria of the observation instrument. An *ad hoc* observation tool was developed to be used subsequently in HOISAN software, in which seven bouts were viewed, collecting the data on the participants individually (14 viewings).

### Participants

The study sample comprised a total of seven contests, consisting of the finals of the Roma 2019 Grand Prix (women, *n* = 3; men, *n* = 4). Women: Fly −49 Kg (Russia vs. South Korea), Feather −57 Kg (South Korea vs. Turkey), Welter −67 Kg (Croatia vs. South Korea). Men: FLY −58 Kg (South Korea vs. Spain), Feather −68 Kg (Iran vs. South Korea), Welter −80 Kg (Russia vs. Spain), Heavy +80 Kg (Russia vs. Kazakhstan).

A total of 1,382 technical-tactical actions were compiled (women, *n* = 687; men, *n* = 695). One women's final did not take place because one of the participants was injured. The number of viewings was estimated on the basis of a generalizability analysis (Blanco-Villaseñor et al., 2014). According to the rules laid down by the Ethics Committee and by the Belmont report (National Commission for the Protection of Human Subjects of Biomedical Behavioural Research, 1978) the competitors' informed consent was not necessary, since they were public videos viewed on a website (YouTube) and did not involve any kind of experimentation (López-López et al., 2015).

### Instruments

A mixture of field format and category systems was used as an observation instrument, following previous studies (Falcó et al., 2014; Menescardi et al., 2015, 2020b). Each category was exhaustive and mutually exclusive for each criterion. This instrument (Table 1) is composed of five criteria (Tactical action, Type of technique, Strike area and Sidedness, Scoring) and 30 categories. HOISAN software was used for coding the athletes' actions (Hernández-Mendo et al., 2012a, 2014). It allows coding, analysis, and calculation and vectorial representation of polar coordinates (López-López et al., 2015).

**Table 1:** Observation tool and coding system.

**Criterion**	**Code**	**Category**	**Category definition**
Tactical action	DA	Direct attack	Offensive move not preceded by any prior movement and performed with the intention of scoring.
	IA	Indirect attack	Offensive move preceded by a prior movement (change of position, jump, etc.) and performed with the intention of scoring.
	CORR	Correction	Move combining two technical actions executed with the same leg or different legs.
	MEL	Melee	Attacking action in which the opponents are at very close range.
	SUBCA	Subsequent counterattack	Defensive move initiated after the opponent's attacking action.
	SIMCA	Simultaneous counterattack	Defensive move initiated at the same time as the opponent's attacking action.
	ANTCA	Anticipatory counterattack	Defensive move that anticipates the attacking action initiated by the opponent.
	CLA	Clash	Defensive action attempting to impede the opponent's attack using the legs.
	BLO	Block	Move performed with the hands, used when the attack cannot be dodged.
	DOD	Dodge	Backward movement to evade the opponent's attack.
Type of technique	LIN	Linear	Attack executed with a straight kick.
	CIR	Circular	Attack executed with a kick involving a circular movement of the hip or knee.
	TUR	Turning	Attack executed with a 180° or 360° turn.
	PUN	Punch	Strike delivered with the hand closed into a fist.
	SING	Single	Corrective action, performed with the same leg, following an up-down or down-up trajectory.
	DOU	Double	Corrective action, performed with different legs.
	NOT	None	No technique executed.
Strike area	HEA	Head	Strike to the permitted areas of the head. In the case of a single or double correction, the head will be considered the last action.
	TRU	Trunk	Strike to the permitted areas of the trunk. In the case of a single or double correction, the trunk will be considered the last action.
	NOA	None	No strike area.
Sidedness	RT	Right	Technique executed from the right side of the body.
	LF	Left	Technique executed from the left side of the body.
	NEU	Neutral	Kick or punch executed from an imperceptible angle of view, also present in dodging and blocking.
Scoring	0P	Zero points	Ineffective attack with the first or foot.
	1P	One point	Valid punch to the trunk.
	2P	Two points	Valid kick to the trunk.
	3P	Three points	Valid kick to the head.
	4P	Four points	Valid turning kick to the trunk or achieving a correction to the trunk only.
	5P	Five points	Valid turning kick to the head or achieving a correction to the trunk and head.
	GJ	Gam-jeom	Action entailing a penalty or warning (crossing the boundary line, falling down, avoiding the match, attacking the fallen opponent, etc.) and signaled by the referee with a horizontal movement of the arm. This action gives one point to the opponent.

### Procedure

The process involved three observers with the support of an expert observer. The observer training and coaching phase (Anguera, 2003) determined the observation design, sampling plan, instrument development, coding system and recording. A consensus agreement (Anguera, 1990, 2003) was carried out to reach an agreement prior to recording.

Subsequently, two observers recorded the data of the Fly −58 kg bouts (South Korea vs. Spain) in the men's category and Welter −67 kg (Croatia vs. South Korea) in the women's category. After 15 days, the same bouts were recorded again, obtaining the results of intra-observer agreement. Agreements were established with the other two observers to observe and record the same bouts, obtaining the results of the interobserver concordance. The values obtained in the correlation coefficients (Table 2) and Cohen's kappa indexes (Table 3) were used to verify the reliability of the observational data.

**Table 2:** Interobserver and intraobserver correlation coefficient.

**Coefficient**	**Interobserver agreement**		**Intraobserver agreement**	
	**1 and 2**	**1 and 3**	**2 and 3**	
Pearson	0.99	0.99	0.99	0.99
Spearman	0.99	0.97	0.99	0.99
Kendall tau-b	0.96	0.94	0.98	0.98

**Table 3:** Cohen's kappa indexes for agreement between observers.

**Criteria**	**Interobserver agreement**			**Intraobserver agreement**
	**1 and 2**	**1 and 3**	**2 and 3**	
Complete session	0.93	0.91	0.97	0.99
Tactical action	0.92	0.89	0.96	0.97
Type of technique	0.81	0.72	0.86	1
Strike area	0.87	0.87	1	1
Sidedness	0.85	0.85	1	1
Scoring	0.69	0.69	1	1

Having passed this step, a Generalizability analysis (Cronbach et al., 1972; Cardinet et al., 1976, 1981) was performed following the study by Blanco-Villaseñor et al. (2014). The computer software SAGT v.1.0 (Hernández-Mendo et al., 2012b) was used to verify the interobserver reliability, the homogeneity of the categories and to estimate the minimum number of sessions needed to generalize accurately.

Finally, five focal behaviors (1P, 2P, 3P, 4P, 5P) were selected for the sequential and polar coordinate analyses with HOISAN software (Hernández-Mendo et al., 2012a, 2014) and the graphic representations of the vectors were optimized with the R program (Rodríguez-Medina et al., 2019). Relationships with a vector length of 1.96 or more, based on the characterization of each quadrant proposed by Hernández-Mendo and Anguera (1999), were considered significant (*p* < 0.05): in quadrant I, behaviors with a mutually excitatory relationship between the focal and conditioned behaviors in a prospective perspective and a retrospective perspective; in quadrant II, inhibitory relationships in a prospective perspective and excitatory relationships in a retrospective perspective; in quadrant III, inhibitory relationships in prospective and retrospective perspectives; and finally, in quadrant IV, inhibition relationships in a retrospective perspective and activation relationships in a prospective perspective.

### Statistical Analysis

The generalizability analysis was performed with two facets: categories (C) and observers (O); the Category/Observer (C/O) model was used to test reliability and the Observer/Category model for validity (Table 4), using SAGT v1.0 software (Hernández-Mendo et al., 2012b). It was determined that with two observations a Relative and Absolute Generalizability Coefficient of 0.92 is obtained, and the observation of five bouts guarantees a 0.98. To certify the reliability of the observations, Pearson's, Spearman's and Kendall's tau-b correlation coefficients and Cohen's kappa agreement index were used. In this way, the validity and accuracy of both the tool and the observers were confirmed and accepted; the values were adequate, as they approached or exceeded 0.70 (Blanco et al., 2000).

**Table 4:** Relative and absolute generalizability coefficients.

	**C/O**	**O/C**
Relative	0.926	0.967
Absolute	0.926	0.967

## Results

The following section shows the results of the polar coordinate analysis of the selected one-point (1P), two-point (2P), three-point (3P), four-point (4P), and five-point (5P) focal behaviors and the behavioral maps obtained for winners and losers.

The results for the one-point (1P) focal behavior in winners showed significant relationships in three quadrants (see Table 5). In quadrant I, mutually excitatory relationships were obtained with the correction (CORR) and direct attack (DA) actions, with a high intensity of 4.95. The other associations were with the circular (CIR), single (SING), and double (DOU) techniques and with strikes to the head area (HEA). Quadrant III presented mutual inhibition effects with the indirect attack (IA), block (BLO), clash (CLA), and simultaneous counterattack (SIMCA) conditioned behaviors and with the linear technique (LIN). In losers, the results obtained for the one-point (1P) focal behavior showed relationships with subsequent counterattack (SUBCA), right sidedness (RT), and circular technique (CIR) behaviors in quadrant I; the last of these was also found in winners. In quadrant IV, a significant association of inhibition was found in the retrospective perspective and of activation in the prospective perspective with the simultaneous counterattack (SIMCA) behavior.

**Table 5:** Results of the polar coordinate analysis for the 1P focal category in winners and losers.

	**Winners**					**Losers**				
**Category**	**Quadrant**	**ProspP**	**RetrospP**	**Radius**	**Angle**	**Quadrant**	**ProspP**	**RetrospP**	**Radius**	**Angle**
DA	I	3.54	3.45	4.95*	44.28	IV	0.02	−0.55	0.55	272.17
IA	III	−2.31	−0.81	2.45*	199.3	I	0.35	0.74	0.82	64.5
BLO	III	−1.67	−1.22	2.06*	216.09	IV	0.61	−1.01	1.18	301.2
MEL	II	−0.56	1.07	1.21	117.6	II	−1.35	0	1.35	179.81
ANTCA	III	−0.63	−0.64	0.89	225.53	IV	0.86	−0.11	0.87	353.01
CLA	III	−0.89	−2.57	2.72*	250.93	II	−0.5	0.65	0.82	127.83
CORR	I	3.12	1.4	3.42*	24.11	IV	0.23	−0.4	0.47	299.87
SUBCA	IV	1.52	−0.11	1.52	355.98	I	1.78	1.87	2.58*	46.43
SIMCA	III	−0.76	−1.91	2.06*	248.32	IV	2.25	−0.68	2.35*	343.14
DOD	II	−1.02	0.92	1.37	137.78	III	−1.28	−0.65	1.44	206.74
RT	IV	1.39	−1.31	1.91	316.74	I	1.29	1.87	2.27*	55.4
LF	II	−0.28	1.35	1.38	101.93	III	−0.53	−1.22	1.33	246.42
NEU	III	−1.21	−0.23	1.23	190.74	III	−0.94	−0.89	1.29	223.34
CIR	I	0.41	2.17	2.21*	79.31	I	0.67	2.53	2.62*	75.06
DOU	I	3.83	2.8	4.74*	36.16	IV	0.19	−0.93	0.95	281.4
TUR	II	−0.51	1.01	1.13	116.81	III	−0.44	−0.32	0.54	216.58
LIN	III	−0.75	−2.08	2.21*	250.24	IV	0.52	−0.83	0.98	302.19
NOT	III	−1.15	−0.16	1.16	187.85	III	−0.78	−0.97	1.24	231.32
PUN	I	0.22	0.17	0.28	37.83	II	−0.57	0.67	0.88	130.41
SING	I	2.07	0.15	2.08*	4.08	I	0.69	0.69	0.97	44.89
HEA	I	0.72	2.15	2.26*	71.48	I	0.21	0.29	0.36	54.9
NOA	III	−1.08	−0.09	1.09	184.56	III	−0.71	−0.91	1.15	231.85
TRU	IV	0.47	−1.31	1.4	289.71	I	0.57	0.72	0.92	51.4

*Indicates the significant vectors (radius > 1.96). In addition, the category transitions and prospective and retrospective Zsum values, vector quadrant, radius length, and vector angle are shown for both winners and losers.

**Figure 1:**
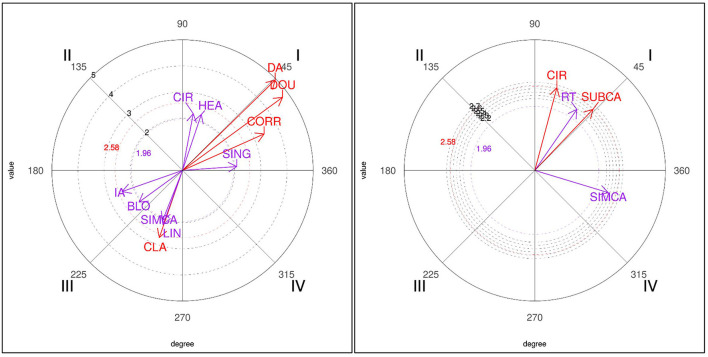
Representation of the behavioral map for the 1P focal behavior in winners (left) and losers (right).

Graphic representations or polar coordinate maps for the 1P focal behavior for both losers and winners are shown in Figure 1.

The two-point (2P) focal behavior showed a lower number of significant relationships with the conditioned categories (see Table 6). In winners, the only mutually excitatory association in the first quadrant was with the circular technique (CIR). In the second quadrant, two associations were found, with the punch technique (PUN) and strike to the trunk area (TRU) conditioned behaviors. In losers, only one significant relationship, corresponding to the first quadrant, was found with the punch technique (PUN), with a high vector length of 3.48.

**Table 6:** Results of the polar coordinate analysis for the 2P focal category in winners and losers.

	**Winners**					**Losers**				
**Category**	**Quadrant**	**ProspP**	**RetrospP**	**Radius**	**Angle**	**Quadrant**	**ProspP**	**RetrospP**	**Radius**	**Angle**
DA	II	−0.4	1.21	1.28	108.35	II	−1.21	1.16	1.68	136.12
IA	IV	0.24	−0.17	0.3	324.99	IV	0.31	−0.52	0.61	300.63
BLO	III	−0.32	−1.63	1.66	258.94	IV	1.02	−1.35	1.69	306.97
MEL	III	−0.22	−0.68	0.71	252.02	I	0.9	0.9	1.27	44.91
ANTCA	III	−0.47	−0.47	0.67	225	II	−0.55	1.32	1.43	112.43
CLA	II	−0.13	0.11	0.17	140.08	IV	0.38	−0.51	0.64	306.3
CORR	I	0.02	1.53	1.53	89.28	II	−0.85	0.38	0.93	156.04
SUBCA	IV	1.8	−0.23	1.81	352.71	II	−0.31	0.62	0.69	116.73
SIMCA	I	1.56	0.07	1.56	2.67	I	0.69	0.7	0.99	45.28
DOD	III	−1.21	−0.31	1.25	194.57	III	−0.29	−1.43	1.46	258.65
RT	I	0.5	0.13	0.51	15.05	II	−0.32	0.86	0.92	110.31
LF	II	−0.06	1.13	1.13	92.82	I	0.8	0.8	1.13	44.95
NEU	III	−0.5	−1.59	1.67	252.66	III	−0.48	−1.86	1.92	255.68
CIR	I	0.73	2.71	2.81*	74.95	II	−0.83	1.32	1.56	122.05
DOU	I	0.86	1.04	1.35	50.34	II	−0.47	1.69	1.75	105.64
TUR	II	−0.99	0.05	0.99	177.08	III	−0.64	−0.6	0.88	223.31
LIN	IV	0.21	−1.86	1.88	276.43	IV	1.08	−1	1.47	317.19
NOT	III	−0.45	−1.55	1.61	253.94	III	−0.39	−1.8	1.84	257.62
PUN	II	−0.03	2.12	2.12*	90.94	I	0.26	3.47	3.48*	85.76
SING	II	−0.44	1.21	1.29	110.1	III	−0.58	−0.58	0.82	225
HEA	IV	0.69	−1.26	1.44	298.51	II	−1.13	0.79	1.38	144.86
NOA	III	−0.39	−1.5	1.55	255.3	III	−0.36	−1.77	1.81	258.45
TRU	II	−0.1	2.12	2.12*	92.83	I	0.88	1.29	1.56	55.66

*Indicates the significant vectors (radius > 1.96). In addition, the category transitions and prospective and retrospective Zsum values, vector quadrant, radius length, and vector angle are shown for both winners and losers.

**Figure 2:**
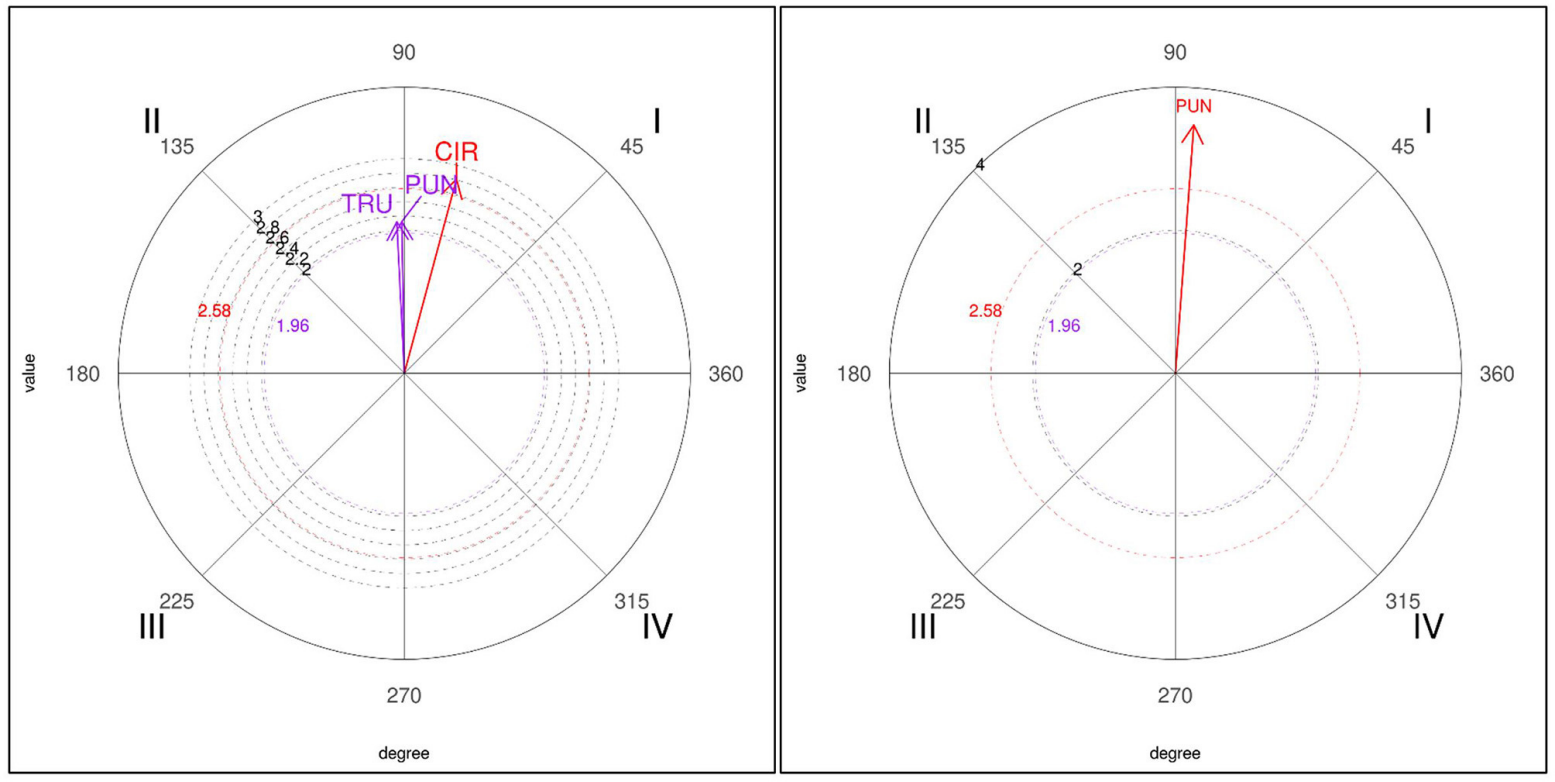
Representation of the behavioral map for the 2P focal behavior in winners (left) and losers (right).

Graphic representations of polar coordinate maps for the 2P focal behavior for both losers and winners are shown in Figure 2.

The results obtained for the three-point (3P) focal behavior (see Table 7) show three significant relationships for winners in quadrant I, with the melee attack (MEL), the punch technique (PUN) and strikes to the head (HEA). In quadrant II, the focal behavior was significantly associated with left sidedness (LF) and anticipatory counterattack (ANTCA); the latter reached an intensity of 3.86 in the relationship. Quadrant IV, on the other hand, showed a relationship with right sidedness (RT). In losers, the results revealed an association with circular techniques in quadrant I, with strikes to the head in quadrant II and with the linear technique in quadrant III.

**Table 7:** Results of the polar coordinate analysis for the 3P focal category in winners and losers.

	**Winners**					**Losers**				
**Category**	**Quadrant**	**ProspP**	**RetrospP**	**Radius**	**Angle**	**Quadrant**	**ProspP**	**RetrospP**	**Radius**	**Angle**
DA	I	0.37	0.04	0.37	6.74	IV	0.73	−0.28	0.78	338.83
IA	III	−1.04	−1.45	1.78	234.5	I	0.3	0.29	0.42	43.87
BLO	III	−0.32	−0.32	0.45	224.84	IV	1.82	−0.55	1.9	343.09
MEL	I	1.15	2.52	2.77*	65.48	I	0.24	0.9	0.93	74.82
ANTCA	II	−0.47	3.83	3.86*	97.03	III	−0.55	−0.55	0.77	225
CLA	III	−0.75	−0.51	0.91	214.01	II	−1.4	0.38	1.45	164.96
CORR	I	0.02	0.04	0.04	63.43	III	−0.85	−0.85	1.2	225
SUBCA	III	−0.23	−0.9	0.93	255.87	IV	0.58	−1.18	1.31	295.96
SIMCA	II	−0.67	1.56	1.7	113.18	II	−0.72	0.7	1.01	136.04
DOD	IV	1.48	−1.21	1.91	320.67	II	−0.29	0.32	0.43	132.29
RT	IV	0.18	−2.11	2.12*	274.81	II	−1.53	0.06	1.53	177.8
LF	II	−0.91	2.27	2.45*	111.92	IV	1.25	−0.53	1.35	337.1
NEU	IV	0.96	−0.49	1.08	332.83	I	0.46	0.5	0.68	47.01
CIR	IV	0.73	−0.76	1.05	313.67	I	2.75	0.61	2.82*	12.41
DOU	IV	0.86	−0.63	1.07	323.43	III	−0.47	−0.47	0.67	225
TUR	II	−0.99	0.05	0.99	177	III	−0.64	−0.6	0.88	223.31
LIN	II	−1.56	0.2	1.57	172.77	III	−2.17	−1	2.39*	204.67
NOT	IV	1.02	−0.81	1.31	321.6	I	0.55	0.11	0.56	11.37
PUN	I	1.4	2.12	2.54*	56.51	I	1.06	1.06	1.5	45.06
SING	II	−0.44	0.39	0.59	138.87	II	−0.58	1.18	1.32	116.07
HEA	I	1.18	1.69	2.07*	55.16	II	−1.13	1.75	2.08*	122.9
NOA	IV	1.08	−0.76	1.33	324.86	I	0.59	0.15	0.61	13.98
TRU	III	−1.7	−0.44	1.76	194.49	III	−0.02	−0.97	0.97	269.07

*Indicates the significant vectors (radius > 1.96). In addition, the category transitions and prospective and retrospective Zsum values, vector quadrant, radius length, and vector angle are shown for both winners and losers.

**Figure 3:**
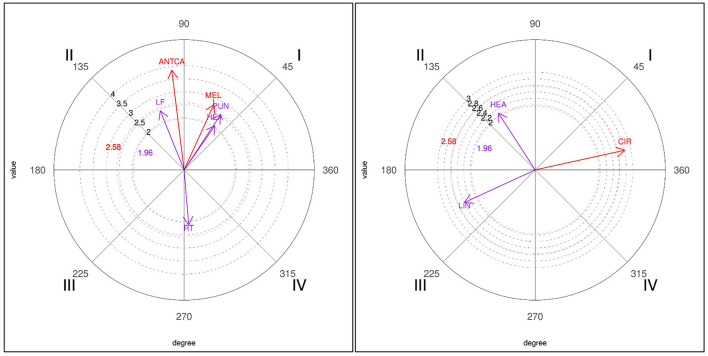
Representation of the behavioral map for the 3P focal behavior in winners (left) and losers (right).

Graphic representations of the polar coordinates for the 3P focal category for both losers and winners are shown in Figure 3.

The data obtained for the four-point (4P) focal behavior showed significant relationships with conditioned behaviors only in winners (see Table 8). In quadrant I, there was a mutually excitatory association between the 4P behavior and the direct attack (DA) tactical action and right sidedness (RT) in prospective and retrospective perspectives. In quadrant II, a highly significant relationship of inhibition in a prospective perspective and excitation in a retrospective perspective was obtained with simultaneous counterattack (SIMCA), with a vectorization of 4.26. There was mutual inhibition with left sidedness (LF) in quadrant III and with the single (SING) technique in quadrant IV.

**Table 8:** Results of the polar coordinate analysis for the 4P focal category in winners.

	**Winners**				
**Category**	**Quadrant**	**ProspP**	**RetrospP**	**Radius**	**Angle**
DA	I	2.68	0.26	2.69*	5.53
IA	III	−0.86	−0.86	1.21	224.93
BLO	III	−0.51	−0.51	0.72	225
MEL	III	−0.79	−0.79	1.11	225
ANTCA	III	−0.15	−0.15	0.21	225
CLA	III	−1.49	−0.55	1.59	200.04
CORR	IV	1.84	−0.45	1.9	346.31
SUBCA	III	−0.5	−0.5	0.7	225
SIMCA	II	−0.44	4.24	4.26*	96
DOD	I	0.61	0.6	0.86	44.89
RT	I	2.68	1.65	3.15*	31.63
LF	III	−2.44	−1.53	2.88*	212.1
NEU	I	0.07	0.07	0.1	44.48
CIR	II	−0.71	0.85	1.11	129.6
DOU	III	−0.21	−0.2	0.29	223.5
TUR	III	−0.31	−0.31	0.44	225
LIN	III	−0.12	−1.05	1.05	263.55
NOT	I	0.09	0.09	0.13	44.58
PUN	II	−0.46	1.8	1.86	104.41
SING	IV	2.19	−0.4	2.23*	349.71
HEA	IV	0.84	−0.71	1.1	319.7
NOA	I	0.11	0.11	0.15	44.65
TRU	II	−0.64	0.36	0.73	150.18

*Indicates the significant vectors (radius > 1.96). In addition, the category transitions and prospective and retrospective Zsum values, vector quadrant, radius length, and vector angle are shown for both winners and losers.

**Figure 4:**
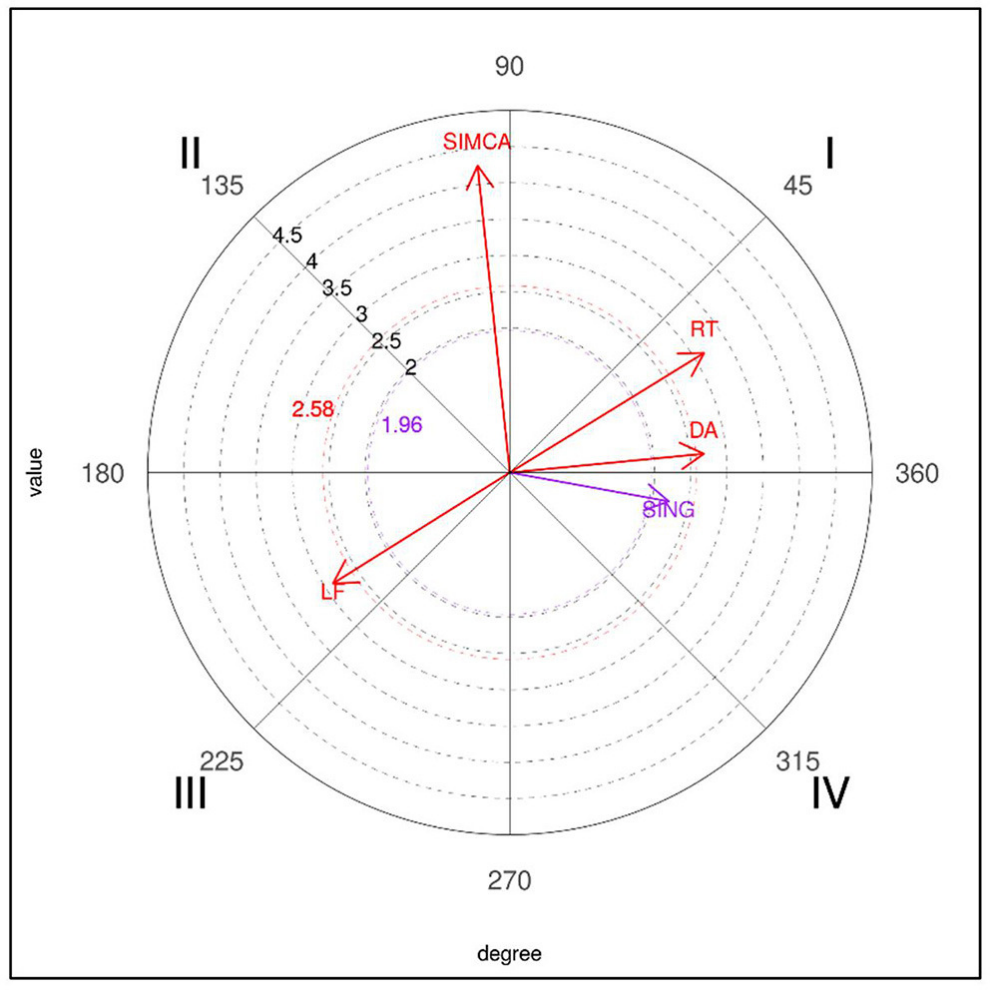
Representation of the behavioral map for the 4P focal behavior in winners.

A graphic representation of the polar coordinates for the 4P focal category for winners is shown in Figure 4.

Similarly, the results obtained for the five-point (5P) focal behavior did not produce any significant associations in losers. In winners (see Table 9), the first quadrant was significantly associated with the circular technique (CIR), generating a radius of 4.64, and also with right sidedness (RT) and direct attacks (DA). The second quadrant showed a relationship with the turning (TUR) technique, the third quadrant with left sidedness (LF), the linear technique (LIN) and the clash (CLA) tactical action, and the fourth quadrant revealed a single significant relationship with the punch technique (PUN), which reached an intensity of 4.08.

**Table 9:** Results of the polar coordinate analysis for the 5P focal category in winners.

		**Winners**			
**Category**	**Quadrant**	**ProspP**	**RetrospP**	**Radius**	**Angle**
DA	I	2.67	0.26	2.69*	5.53
IA	I	0.48	0.48	0.68	45.13
BLO	III	−0.51	−0.51	0.72	225.00
MEL	III	−0.79	−0.79	1.11	225.00
ANTCA	III	−0.15	−0.15	0.21	225.00
CLA	III	−1.49	−1.51	2.12*	225.30
CORR	III	−0.45	−0.45	0.64	224.66
SUBCA	II	−0.5	1.62	1.7	107.00
SIMCA	II	−0.44	1.89	1.94	103.23
DOD	I	0.61	0.61	0.86	45.00
RT	I	1.67	1.65	2.35*	44.70
LF	III	−1.54	−1.53	2.18*	224.78
NEU	I	0.07	0.07	0.11	45.00
CIR	I	2.41	3.97	4.64*	58.75
DOU	III	−0.21	−0.20	0.29	223.50
TUR	II	−0.31	2.97	2.98*	95.99
LIN	III	−2.89	−2.90	4.1*	225.05
NOT	I	0.09	0.09	0.13	45.00
PUN	IV	4.06	−0.46	4.08*	353.50
SING	III	−0.40	−0.40	0.56	225.00
HEA	I	0.83	0.85	1.19	45.56
NOA	I	0.11	0.11	0.16	45.00
TRU	III	−0.64	−0.64	0.9	225.22

*Indicates the significant vectors (radius > 1.96). In addition, the category transitions and prospective and retrospective Zsum values, vector quadrant, radius length, and vector angle are shown for both winners and losers.

**Figure 5:**
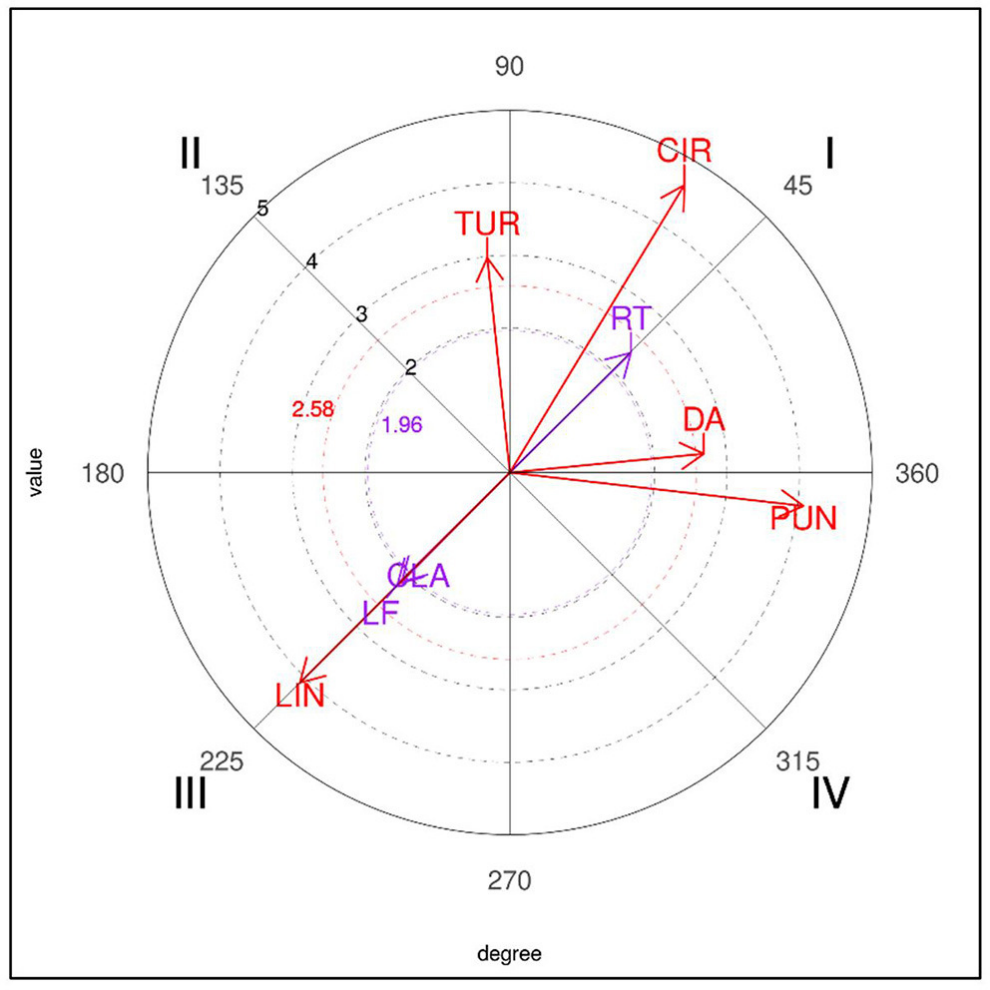
Representation of the behavioral map for the 5P focal behavior in winners.

A graphic representation of the polar coordinates for the 5P focal category for winners is shown in Figure 5.

## Discussion

The objective of this study was to design, validate and update an observation tool and use it to analyse the technical-tactical actions by which taekwondo players win points, comparing winners and losers of the finals of the Rome 2019 Grand Prix in this respect. In pursuit of this objective, a generalizability analysis was first performed using the C/O and O/C models (Table 4) for validity and the Pearson, Spearman, Kendall's tau-b, and Cohen's kappa coefficients for reliability; the values were appropriate, as they were higher than or close to 0.70 (Blanco et al., 2000). This was followed by a polar coordinate analysis, taking as focal behaviors those related to obtaining points (1P, 2P, 3P, 4P and 5P) and as conditioned behaviors those related to technical-tactical actions: direct attack (DA), indirect attack (IA), correction (CORR), melee (MEL), subsequent counterattack (SUBCA), simultaneous counterattack (SIMCA), anticipatory counterattack (ANTCA), clash (CLA), block (BLO), dodge (DOD), linear technique (LIN), circular technique (CIR), turning technique (TUR), punch (PUN), single corrective action (SING), double corrective action (DOU), strike to the helmet (HEA), to the trunk protector (TRU), with the right leg (RT) or the left leg (LF) or in a neutral position when in a melee (NEU).

With regard to the 1P focal behavior (obtaining one point with a valid punch to the trunk protector or by a penalty given against the opponent), the results show different patterns in winners and losers. In winners, obtaining a point was preceded and followed by direct attack and correction behaviors, with a single or double technique, using a circular kick to the head (quadrant I). In losers, obtaining a point was preceded and followed by a subsequent counterattack, with a circular kick executed with the right leg. It is important to emphasize that one point is scored by a punch or by a penalty given against the opponent. In this regard, it is reasonable to think that as a consequence of the actions performed, a penalty is awarded against the opponent, adding a point to the score. The results of our study are in line with previous studies (Kazemi et al., 2006; González-Prado et al., 2015) which show that losers incur more penalties than winners.

It is also interesting to highlight the occurrence of single or double correction actions, which, even if they do not succeed in scoring, do give rise to an action by the opponent that incurs a penalty. Furthermore, the occurrence of these behaviors reveals the tactical evolution of the sport. This is the first study that includes these variables (single and double correction, as well as clash), and therefore more studies showing the effectiveness of such technical-tactical actions are needed. It is worth emphasizing that the fact that winners provoke a penalty through direct attack and correction behaviors, whereas losers obtain it through a subsequent counterattack, is in line with previous studies (González-Prado et al., 2015) showing that the more offensive actions are executed, the more points are scored, and contrary to other studies showing that winners carry out more counterattacks than losers (Falcó et al., 2014; Menescardi et al., 2015) or perform more anticipatory actions (obtaining one point), while losers execute a higher number of back-leg, indirect and turning actions (Menescardi et al., 2020c). Regarding inhibited (neutralized) actions in winners, these are indirect attack, simultaneous counterattack, block, clash, and linear technique (quadrant III), whereas inhibited behaviors are not observed in losers, which presumably reveals the active attacking style in winners.

The 2P focal behavior (obtaining two points by means of a valid kick to the trunk protector, or scoring one point for a punch plus a penalty given against the opponent) shows a similar behavioral pattern in winners and losers, since both groups use the punch to obtain a point, while the opponent receives a penalty at the same time (and therefore they obtain two points in the action). It is an uncommon score both in winners and in losers. According to studies that refer to it (González-Prado et al., 2015) it is a difficult action to score, but is executed in this case in order to continue adding after obtaining one point and not as a counterattacking or defensive action to evade the opponent, while in winners the circular behavior is found. Previous studies that use a different scoring system, but that have similarly analyzed obtaining points by executing a linear or circular kick to the trunk protector, have shown that effective actions are performed with direct strikes to the trunk and that after scoring, players continue with an action to the helmet (Menescardi et al., 2019) or execute a kick with the back leg or a cutting action (Menescardi et al., 2020a). Other studies show that winners perform more anticipatory actions (managing to score), whereas losers execute more rear-leg indirect actions (Menescardi et al., 2020c). The lack of two-point scores could be due to better blocking by taekwondo athletes, on the one hand, and to the difficulty of scoring on the trunk protector, owing to their power threshold levels (Daedo, 2020).

For the 3P focal behavior (obtaining three points by a valid kick to the helmet), winners show the melee, punch and head strike conditioned behaviors in quadrant I, while losers perform circular actions. Prior to scoring three points (quadrant III), winners execute anticipatory actions with the left leg. These results are in line with previous studies (Menescardi et al., 2020c) which show the use of anticipatory actions by winners to obtain three points, because of their effectiveness (Falcó et al., 2014). The range of possibilities that winners display to obtain three points is interesting, as they do so either with a melee action, a punch action followed by another melee, an anticipatory action with the left leg followed by a melee action, a punch or another technique with the right leg (quadrant IV). Once again we can see how tactical action has evolved, since previous studies have shown that to obtain three points winners execute more anticipatory actions (Menescardi et al., 2020c), indirect attacks or subsequent counterattacks to the helmet (Menescardi et al., 2019) cutting actions occur before scoring (Menescardi et al., 2019, 2020b).

Finally, in relation to the 4P behavior (obtaining four points for a valid turning kick to the trunk protector) and the 5P behavior (obtaining five points for a valid kick to the helmet preceded by a turn), it should be noted that they were only performed by winners. According to some studies (Falcó et al., 2014; Menescardi et al., 2019) very specific actions must be executed to achieve these scores: not being averse to failing, and taking account of the time needed to carry it out. To score, both are performed in a direct attack with the right leg (the left is inhibited), with turning (in the case of scoring five points) or simultaneous (in the case of four points). In previous studies, when two points were obtained (instead of four) with actions preceded by turning, they were achieved with simultaneous turning actions, followed by subsequent counterattacks, whereas cutting actions occurred before and after scoring (Menescardi et al., 2019). Three points (instead of five) were obtained with a turning kick to the head, continuing with an action with the front leg (Menescardi et al., 2020b). As has been suggested (Menescardi et al., 2020b), it seems that winners are capable of using more complex actions, and by doing so they achieve higher scores which enable them to win the contest.

## Conclusion and Pratical Applications

Four conclusions and practical applications can be drawn from this study in relation to how to score in a taekwondo bout:

1. With regard to obtaining one point (for a valid punch to the trunk protector or for a penalty given against the opponent), both winners and losers do so by the opponent being penalized. It should note that in winners this action is preceded and followed by a direct attack or correction, a single or double technique or a circular kick to the head. In losers, it is preceded and followed by a subsequent counterattack, with a circular kick executed with the right leg.
2. Two points are obtained for a valid circular kick to the trunk protector (in winners), or by scoring a point for a punch plus a penalty against the opponent (in both winners and losers).
3. Three points (obtained with a valid kick to the helmet) are observed in winners in melee actions or after a punch, whereas in losers they occur in circular actions. Beforehand, winners execute either anticipatory actions with the left leg, a melee action, an action consisting of a punch followed by another melee, an anticipatory action with the left leg followed by a melee or punch action, or another kick with the right leg.
4. Behaviors obtaining four points (by a valid turning kick to the trunk protector) and five points (by a valid kick, preceded by a turn, to the helmet) are only performed by winners. To do so, they execute a direct attack with the right leg (the left is inhibited), with turning (in the case of scoring five points) or simultaneous (in the case of obtaining four points).

## Limitations and Future Lines of Research

As regards the limitations of this study, the results obtained are significant and provide athletes and coaches with the necessary information. Additional studies should consider increasing the number of observations to provide a larger number of patterns. In this study, the planned sample was the eight final bouts of the Grand Prix of Rome 2019 (four female and four male bouts). The female HEAVY +67 kg final was not held due to the injury of one of the participants in that category, limiting the observation and recording of that bout. The data obtained did not affect the results of the study, since the generalizability analysis determined that the observation of five bouts guaranteed a Relative and Absolute Generalizability Coefficient of.98. Therefore, the significant relationships obtained in the polar coordinates analysis between the selected focal categories and the rest of the categories of the tool are considered generalizable in the study. As a future line of research it would be interesting to track two of the winners throughout the whole championship.

## Data Availability Statement

The original contributions presented in the study are included in the article/supplementary material, further inquiries can be directed to the corresponding author.

## Author Contributions

GA, AH-M, VM-S, and CF conceived and designed the study. JG-C and YQ-R collected the data and analyzed them. All authors contributed to the interpretation of the results, reviewed and provided feedback to the manuscript, and the final version of the manuscript and approved it for publication.

## Conflict of Interest

The authors declare that the research was conducted in the absence of any commercial or financial relationships that could be construed as a potential conflict of interest.

## Publisher's Note

All claims expressed in this article are solely those of the authors and do not necessarily represent those of their affiliated organizations, or those of the publisher, the editors and the reviewers. Any product that may be evaluated in this article, or claim that may be made by its manufacturer, is not guaranteed or endorsed by the publisher.
